# The leading role of expert safety knowledge in supporting the mission of caring for patients during man-made and natural disasters: state of emergency medicine in Ethiopia, Myanmar, and Ukraine

**DOI:** 10.1186/s12245-024-00609-1

**Published:** 2024-03-01

**Authors:** Ralph C. Miles, Vivian I. Avelino-Silva, Wilfred Odoke, Jan van den Hombergh, Fernanda F. Fonseca, Mengistu GebreMichael, Yaroslava Lopatina, Win Oo, Adele Schwartz Benzaken

**Affiliations:** 1 Aids Healthcare Foundation Global Program, 6255 Sunset Blvd., 21st Fl, Los Angeles, CA 90028 USA; 2 Aids Healthcare Foundation Ethiopia, Addis Ababa, Ethiopia; 3 Aids Healthcare Foundation Ukraine, Kyiv, Ukraine; 4 Aids Healthcare Foundation Myanmar, Yangon, Myanmar

**Keywords:** Disaster Planning, Security measures, Civil Defense, Health Care Facilities, Manpower, and services

## Abstract

Preparedness to endure extreme situations such as natural disasters or military conflicts is not commonplace in healthcare training programs. Moreover, multidisciplinary teams in health services rarely (if ever) include experts in security. However, when emergency situations occur, prevailing healthcare demands do not cease to exist, and unexpected demands often surge due to the shortage of other services and supplies or as a consequence of the emergency condition itself.

With services in 45 countries, AIDS Healthcare Foundation (AHF) has operated in several conflict zones, facing broad and challenging security demands. Since 2017 AHF has implemented the Global Department of Safety and Security (GDSS), a dedicated intelligence and safety program that had a key role in the security monitoring, preparedness, and defense responses, assisting staff members and clients during recent conflicts.

In this manuscript, we describe the experience of AHF’s GDSS in three recent military conflicts in Ethiopia, Myanmar, and Ukraine, and provide insights into steps that can be taken to assure staff safety and support the mission of caring for patients throughout catastrophic events.

## Background

In December 2021, the AIDS Healthcare Foundation (AHF) Global Department of Safety and Security (GDSS) recommended to the overall AHF leadership, Europe Bureau, and country program members that preparations be made for a potential Russian invasion in Ukraine. Such recommendations were not trivial - they affected the daily routine of staff members and more than 50,000 clients in HIV care in the Ukraine program and represented a significant additional expenditure to the institution. The initiative was implemented based on a rigorous monitoring and analysis process of the disintegrating diplomatic ties as well as the observation that Russian military forces were initiating unequivocal offensive movements. After almost two years of unremitting military conflict, the impact of the projected risk assessment and implementation of a preparedness plan is now reflected on the fact that AHF staff and their families have been able to protect themselves and remain in the country, providing services. Moreover, HIV prevention and care indicators highlight some of the concrete effects of this intervention: the AHF program in Ukraine experienced an increase in new enrollments in care, new antiretroviral therapy initiations, and number of clinics in active operation, reflecting the continuity of testing, focus on linkage to care, and an efficient approach to open new facilities supporting both internally displaced clients and patients relocated from other services.

Retrospectively, the decision to prepare the organization for the chaotic impact of a potential conflict seems obvious. Yet, its implementation was only possible due to the expertise of a qualified security and intelligence team with previously built credibility across the institution. Ordinarily, experts in security are not envisioned as part of multidisciplinary teams in healthcare services or health institutions. Similarly, most healthcare training programs do not comprise preparedness for extreme situations such as natural disasters or military conflicts. However, the importance of an intelligence and security strategy cannot be underestimated; a well-developed and thought-out intelligence program has the ability to provide early warnings about threats that can impact critical service operations. Predicting risks even a few days before a catastrophic event happens is crucial to determine one’s ability to protect staff members, clients, assets, and to allow the continuity of health services. Ultimately, when emergency situations occur, prevailing healthcare demands do not cease to exist, and organizations are further challenged with unexpected demands resulting from the shortage of other services and supplies, or from the emergence of novel ailments caused by the disaster itself.

The analysis of risk mostly relies on information available through open sources, decoded by experienced security specialists from a staggering amount of data comprising both useful evidence and lots of noise. Once impending risks are identified, knowledge and background safety expertise are crucial in guiding actions that will determine if a healthcare facility will be able to continue providing services. In other words, security and intelligence are grounded on specific skills and techniques that are unknown to healthcare providers, yet necessary to continue the mission of caring for patients when disasters occur.

AHF is a global non-governmental organization specializing in HIV prevention and care of since 1987. Currently, AHF supports more than 1.8 million clients in care in 45 countries including low, middle, and high-income states. To accomplish the goal of providing the best available care to patients, AHF collaborates with local institutions and governments, combining existing resources with additional services to achieve a tailored, optimized care strategy to each country’s fight against HIV. Working in a wide range of places and conditions means that AHF must have good relationships with all levels of government, maintaining a neutral position towards political conflicts. Simultaneously, a global operation also entails broad and challenging security demands.

Although several international armistice settlements have been established, particularly after World War II, challenges such as social inequalities and deteriorating political relations in the past years indicate with no uncertainty that the civil society continues to face security risks. This led AHF to implement the GDSS in 2017, aiming to enhance the wellbeing of patients, staff members and their families, and to support continuity of care to the best possible extent during extreme situations. This decision quickly proved to be crucial. AHF’s GDSS has been a protagonist in three recent military conflicts in Ethiopia, Myanmar, and Ukraine. Here we describe the challenges and achievements of the program and discuss insights into actions that can be done to guarantee the safety of clients and staff members, and support continuity of care throughout catastrophic events.

### Tigray war (Ethiopia, November 2020 - November 2022)

Ethiopia is the second most populous country in Africa with approximately 114 million inhabitants, of whom 610,000 are estimated to live with HIV. As seen in many countries in Sub-Saharan Africa, HIV prevalence is higher among adult women (1.0%) compared to men (0.6%), and more than 300,000 children and adolescents aged 0–17 are orphans due to Aids. In the past years, Ethiopia registered significant improvements in key HIV indicators, with 70% reduction in new HIV infections and 57% reduction in Aids-related deaths since 2010 [1].

AHF started operations in Ethiopia in 2012, providing comprehensive care services for people living with HIV, including counseling and testing, antiretroviral therapy, treatment of opportunistic infections, diagnosis and management of other sexually transmitted infections, and condom distribution and promotion.

The 1,104,300 km^2^ Ethiopian territory is divided in 11 administrative regions that are home to long-standing ethno-nationalist tensions. In November 2020, the federal defense force started a military offensive against the local dominant group Tigray People’s Liberation Front (TPLF) in the northern region of the country. Over two years, the conflict led to thousands dead and millions displaced [2, 3]. AHF became concerned about the potential impact of the conflict and its harmful effects on staff members and clients in September 2021, following the escalation of Tigrayan forces towards Addis Ababa metropolitan area, where AHF concentrated most of its operations. The preparedness plan included the identification of evacuation routes for staff members, and the development of a communication plan less likely to be interrupted by cyberattacks. AHF conducted training sessions with employees, anticipating the impact of a potential invasion, and prioritizing strategies to shelter in place or prepare for evacuation if needed.

According to testimonials from our local staff, healthcare providers have reported frequent interruptions of HIV services in various facilities located in conflict areas. Patients also reported that they were frequently unable to get their antiretroviral medications, either because they couldn’t visit the facilities due to fear of the ongoing conflicts or because the facilities where they had follow up were destroyed. A number of patients were unable to travel to receive care at higher level health institutions even though they were given referrals; this happened because roads were blocked, means of transportation were unavailable, patients and their family members were unable to afford the travel costs due to the economic impact of the civil war, or because they were not allowed to cross inland borders due to their ethnicity. Hospitals and health units were occupied, looted, and destroyed. Besides the incidents affecting healthcare facilities, providers were victims of violence including episodes of physical injury, sexual assault, and assassinations [4, 5].

The inability to access services or scarcity of medical supplies in functioning facilities has forced Ethiopians in several regions to interrupt treatment and care of multiple chronic conditions, including diabetes, hypertension, heart disease, tuberculosis, and cancer. People who needed tertiary level services experienced delays and many developed complications that could have been avoided had treatment been timely implemented [6].

Although the military conflict officially ended in November 2022, the results of massive displacement and destruction of civilian structures and essential social services over 24 months were catastrophic. The conflict exacerbated food insecurity, sparked outbreaks of infectious diseases such as malaria, measles, and cholera, and collapsed immunization programs and health services [7, 8].

**Fig. 1:**
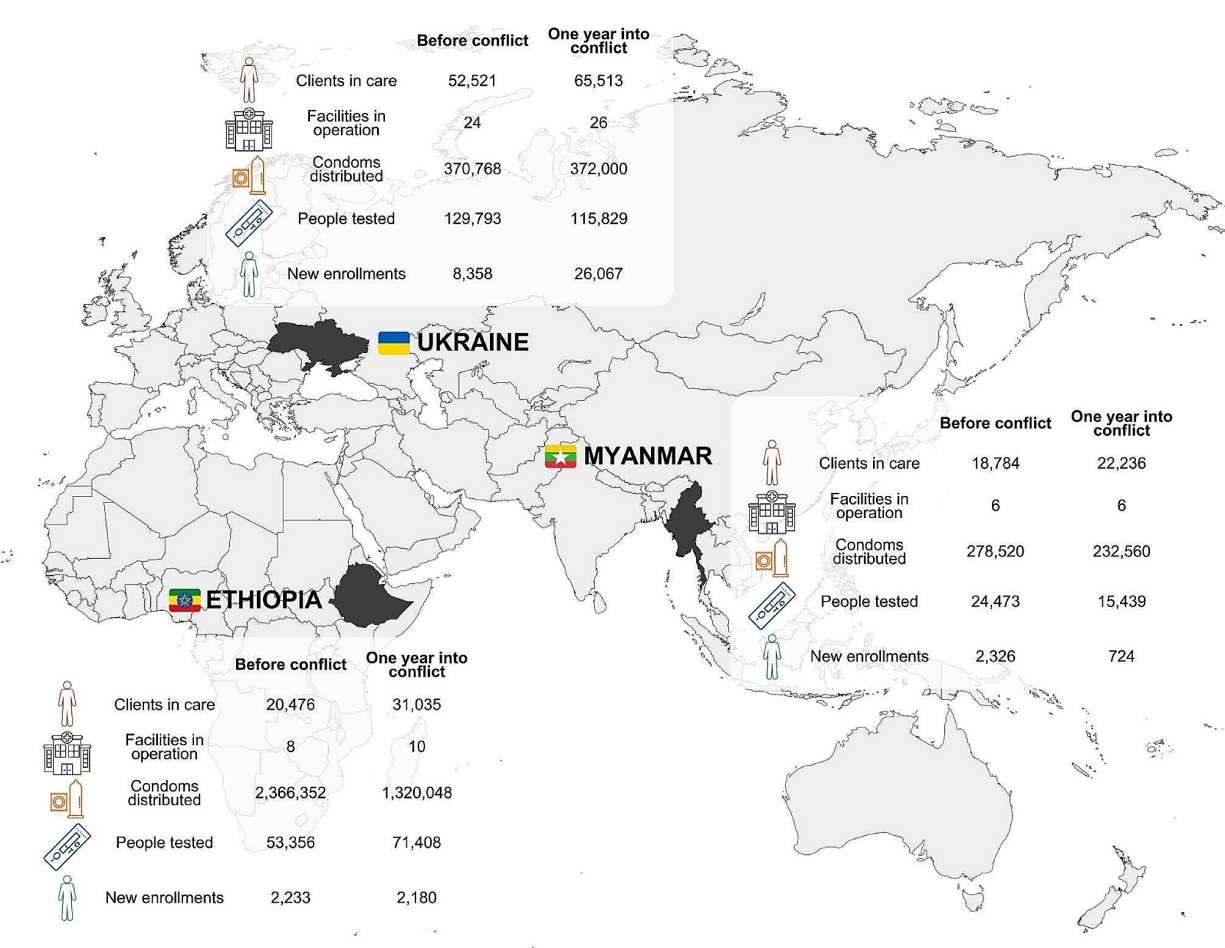
AHF’s HIV prevention and care indicators in Ethiopia, Myanmar, and Ukraine before/after the conflict onset. Figure developed by the authors using MapChart (Version 2.0, Quadratyx, Estonia).

AHF operations were significantly affected during the war in Ethiopia. Although there was no closure of services, the program reported a significant reduction in the number of condoms distributed in the country, and a small reduction in newly enrolled clients one year after the conflict onset compared to the same period one year before. Despite these difficulties, a few indicators demonstrate that the preparedness plan allowed the continuation of key services in AHF facilities throughout this challenging period (Fig. 1). Immediately before the conflict onset in Ethiopia, AHF had 20,476 clients in care; this number increased to 31,035 in October 2021, one year into the conflict, and recent data from July 2023 shows 43,494 clients in care in AHF facilities across the country. The number of facilities under the auspices of AHF also increased since 2020, from eight to twelve units.

### Myanmar coup d’état (Myanmar, February 2021)

Previously known as Burma, Myanmar is a 57 million-inhabitant southeast Asian country, with independence from United Kingdom since 1948 [9]. The country’s history is characterized by intense struggles for ethnic and territorial autonomy. Myanmar’s first free election in 2015 was followed by a coup d’état started on February 1st, 2021, on the day the Parliament was to endorse the election results [10]. Mass demonstrations and conflicts between civil protesters and the military junta arose. So far, the conflict displaced at least 1.4 million people and left a third of the country’s population in need of support [11].

A distinct aspect that further complicated matters in Myanmar was the fact that many healthcare workers were in the front line of the country’s civil disobedience demonstrations [12]. Consequently, security forces arrested providers at their homes, clinics and hospitals, and dozens were killed [13]; Myanmar has been considered one of the most dangerous places in the world to be a healthcare worker [14].

According to UNAIDS data, approximately 280,000 adults and children are living with HIV in Myanmar, with higher prevalence among men (1.0%) compared to women (0.7%). Despite the political adversities, since 2010 the percentage of new HIV infections dropped 35%, while the percentage of Aids-related deaths declined 47% [15].

AHF has been providing overarching and free-of-charge HIV prevention and care services in Myanmar since 2015. AHF’s intelligence and safety program was surprised by the coup despite the historical political instability in Myanmar. The intensification of the conflict prompted concerns about AHF’s staff members and more than 18,000 clients in care. Past interactions with Asia Bureau and country program teams supported the establishment of AHF’s GDSS program’s credibility in conducting the Myanmar crisis response. The GDSS led periodic meetings with key country personnel explaining the events that could be anticipated, the potential impact of attacks, how to avoid them, and how to ensure security of AHF’s assets.

Many healthcare facilities endured shortage of human resources since the military coup, including AHF partner sites. During the first months, hospitals and clinics were temporarily shut down; civil unrest and violence, mobility restrictions, and limited health supplies hampered access to HIV care services. Facilities gradually resumed activities with limited staff and resources. Besides the incidents involving healthcare providers, local facilities reported that some patients were lost to follow-up after being arrested or fleeing to border areas. Mobile testing for HIV and other sexually transmitted infections and community-based education campaigns had to be cancelled due to restrictions on public gatherings. AHF Myanmar focused on sustaining access to HIV testing and treatment during the conflict. Because of workforce shortages, AHF relied on different categories of health personnel and technicians to ensure sustained provision of services, including peer volunteers trained to conduct targeted recruitment for HIV counseling and testing among key affected populations.

Before the military coup d’état, AHF registered 18,784 clients in care in six facilities; of those, five were located in central Myanmar (four in Yangon, one in Bago region), and one was located in Kayin State, at the frontier with Thailand. Over 12 months before the conflict set place, AHF provided diagnostic testing for more than 24,000 people and enrolled more than 2,300 new clients in care. One year after the coup, the program registered drawbacks in the number of condoms distributed in the country, the number of people tested for HIV, and the number of newly enrolled clients; despite these challenges, the total number of clients in care increased 18%, whereas the number of facilities operated by AHF increased from six to eight units (Fig. 1).

### Russian invasion in Ukraine (Ukraine, February 2021)

With independence from former Soviet Union in 1991, Ukraine is the second largest country in eastern Europe after Russia. Most of its 43 million inhabitants are Ukrainians, but a significant minority are Russians, reflecting a long history of occupation and dispute, particularly over the eastern parts of the country and the Crimean Peninsula [16, 17].

Ukraine is home to approximately 240,000 people living with HIV, with key populations including people who inject drugs, prisoners, men who have sex with men, and sex workers being disproportionately affected [18]. AHF started operations in Ukraine in 2010 in collaboration with the Ministry of Health and with regional health departments, offering comprehensive HIV care services at no cost for its clients. In the past 12 years, Ukraine registered a 47% reduction in new HIV infections and 81% reduction in Aids-related deaths [18].

Several months before the Russian invasion, AHF’s GDSS program had identified Ukraine as an incontestable Targeted Area of Interest – a location of specific concern that requires being monitored by a specialist, using an intelligence data collection and assessment cycle. Based on this analysis, the security program was able to predict that the possibility of Russian invasion has very high, even when most people believed that an offensive intervention would not occur. The risk assessment triggered the implementation of safety provisions across all Ukrainian facilities. The overarching plan included staff training, communication and cyber security, basic supplies and financial provisions for staff and their family members, and medical supplies/equipment to prevent interruptions of healthcare services. Preparedness has enabled AHF employees to take care of themselves and their families, allowing the continuation of direct patient care in most sites and even the expansion to new locations despite the challenges imposed by the military conflict.

Since early 2022, health facilities and providers in Ukraine have been facing major challenges, with multiple reports documenting direct impact of the military conflict on healthcare infrastructure and personnel [19–21]. AHF-supported Mariupol AIDS Center was destroyed and became non-operational along with many other civil facilities located in southeast regions of Ukraine occupied by Russian forces over the first months of conflict.

Before the conflict onset, AHF oversaw 24 facilities and had more than 52,000 registered clients in care in the country. After the onset of the military conflict and the destruction of the Mariupol AIDS Center, AHF Ukraine established new partnerships with three governmental institutions located in western regions, aiming to support people living with HIV who were displaced from eastern occupied regions. Remarkably, the number of newly enrolled clients went from 8,359 in the 12 months before the invasion, up to 26,067 in the year following the conflict onset; in 2023, AHF registered 26 facilities and more than 65,000 clients were under the responsibility of the Ukraine program. (Fig. 1). This striking increase in new enrollments resulted largely from admissions of displaced persons and patients whose original health services collapsed during the conflict.

Finally, besides internal displacements, millions of Ukrainians sought refuge in neighboring countries since the beginning of the conflict. Taking advantage of AHF’s global operations, AHF Ukraine and AHF Europe organized a network to support and facilitate linkage to care of Ukrainians living with HIV who needed integration into health services in countries of transition or arrival.

## Conclusion

Emergency situations will inevitably trigger evacuations, service and business closures, economic constraints, and supply shortages, challenging the safety of patients, healthcare workers, and their families. Moreover, extreme circumstances may impact health services operations and continuity of care. Our manuscript describes the experience of AHF’s GDSS program in three recent military conflicts, bringing insights into provisions that can be taken to support providers and clients during emergency situations.

**Fig. 2:**
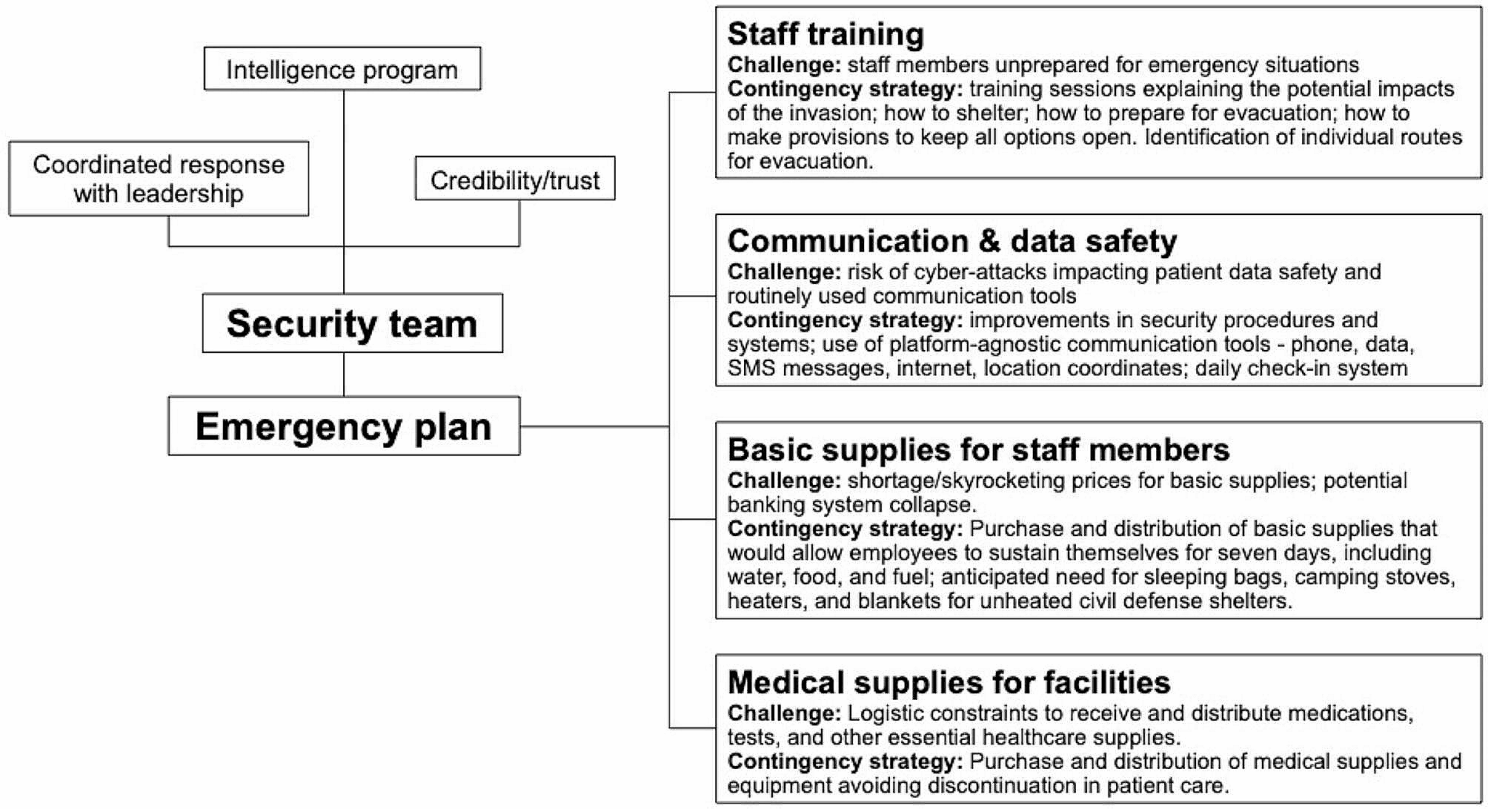
Summary of strategies to be prioritized in the preparedness plan healthcare organizations

Effective preparedness depends on the existence of an experienced intelligence team with established credibility, working closely with leaders and stakeholders to make assertive decisions. The anticipation of risks through the continuous intelligence monitoring and the prompt establishment of an emergency response plan are key to operationalize the timely provision of resources. The components of the response vary according with the type and scale of the anticipated warfare, as well as the characteristics of the conflict zone: at the lower end of the spectrum, warfare may be limited to protests and civil disobedience demonstrations; at the higher end, conflicts may be generalized, with mechanized warfare involving multiple countries. Understanding of the spectrum of actions allows reasonable forecasting of the characteristics of potential attacks, including the types of weaponry, speed and number of combatants, and possible level of destruction. The emergency plan should then match the anticipated risks, including staff training on what to expect from the conflict, how to shelter in place, how to prepare for evacuation; defense infrastructure such as air raid shelters; streamlined communication systems and cyber security; definition and periodic revision of evacuation routes; basic supplies for staff and their family members; and medical supplies/equipment to prevent discontinuation of patient care. On each of these steps, our team has experienced broad and diverse challenges, developing pragmatic solutions (Fig. 2). For instance, attacks to communication resources during extreme situations may block vital messaging systems that are crucially needed to establish and guide evacuation routes; moreover, cyberattacks may compromise access to medical charts and patient confidentiality. Therefore, provisions must be adopted to guarantee alternative communication tools and improve data safety; additionally, a check-in system with daily updates about secure evacuation routes and triggering signs should be implemented during critical periods.

Although AHF’s GDSS program focuses primarily on AHF personnel and assets, multiple elements of the preparedness plan also benefit other local collaborators and partner institutions. For instance, all AHF contractors in Ukraine, including government-hired staff working in AHF-supported facilities, were connected to the communication system that allowed daily reporting AHF’s GDSS about their physical condition, basic supplies availability, and need to evacuate. As another example, AHF supported the renovation of Kyiv Hospital’s basement into a fully equipped 70-person bomb shelter.

Even a well prepared and experienced security program will face challenges and limitations. It may be very difficult to anticipate the occurrence of conflicts when scarce evidence is available for the intelligence analysis, or when events leading to the conflict occur in a very fast pace, as was the case in Myanmar. Moreover, the implementation of the emergency plan requires a solid relationship and credibility built with the institution’s leadership and staff; financial flexibility; and the ability to make adaptations as the conflict evolves. Of note, the structure and operating methods of the institution have central relevance for both the characteristics of the preparedness plan and the ability to maintain local services. In the case of AHF, we work primarily through direct hiring of local human resources, and hiring staff for deployment in foreign countries is not part of the institution’s routine operations. Therefore, during emergency situations, our staff generally desires to stay near their families and communities, and the GDSS contingency plans focus on providing the resources to allow their continuation on site, as long as safety conditions can be provided; evacuations are therefore restricted to extreme situations when measures are seen as insufficient to guarantee the safety of our collaborators. The staff’s safety and desire to stay are the first conditions to allow continuation of services, and expansions very often occur naturally for those that are able to remain functioning throughout extreme events, resulting from the closure of other facilities and enrollment of displaced patients.

Despite unequivocal advantages, most health services and institutions cannot dedicate resources to constitute a formal Intelligence Program, and sometimes the value of these programs is underestimated as catastrophic events are perceived as extremely unlikely. Yet, it is still a good idea to consult expert knowledge and develop a preparedness strategy. Think of it as a health insurance; you don’t plan to get sick, but in case you do, it’s better to be safe than sorry. Preparedness trainings and drills are routinely implemented to protect civilians from the damages caused by fire, earthquakes, and armed intruders; similarly, a minimal set of safety strategies could be proposed to mitigate the impact of catastrophic situations such as military conflicts in healthcare facilities. A few safety elements could be broadly available in health institutions worldwide, including emergency communication tools, basic survival supplies, and an overall plan for sheltering and evacuation with clear trigger indicators. Recent conflicts have also revealed an appalling reality: healthcare services and providers are unfortunately not spared from violence during military conflicts. In fact, the presumption of safety could lead to suboptimal preparedness and even negligence of risk indicators.

Other potential challenge that may interfere with healthcare operations during military or civil conflicts is the interference of ethnic or nationality contentions in the provision of care. For instance, although AHF did not experience situations where local health staff refused to treat people of certain ethnicities or nationalities, there were reported occasions when healthcare workers were forced not to provide health services for casualties who were from certain armed groups. These are dramatic situations with practical and ethical implications that also require dedicated discussions and preparedness.

While man-made and natural disasters are expected to impact health services, there are steps that can be taken to improve staff safety and preserve patient care. Our experience suggests that a comprehensive and flexible preparedness program founded on expert safety knowledge with continuous support for staff members and patients can mitigate the detrimental impact of military conflicts on healthcare.

## Data Availability

All data generated or analyzed during this study are included in this published article.

## Declarations

### Author expertise

Ralph C. Miles is the director of AHF’s Global Department of Safety and Security. He spent nine years serving as an officer in the U.S. Army in various missions around the world before initiating his civilian career. He has accrued more than 25 years of experience in the private sector working with the design, development, and implementation of security and intelligence strategies in a variety of businesses and organizations.
